# Supplementary material for: Clinical Significance of Combined Epithelial–Mesenchymal Transition Markers Expression and Role of Rac1 in Hepatocellular Carcinoma
Source: Int J Mol Sci. 2023 Jan 16;24(2):1765. doi: 10.3390/ijms24021765 (PMC9865966; doi:10.3390/ijms24021765)
Supplement: Supplementary file 1 [file ijms-24-01765-s001.zip › ijms-2073518-supplementary.pptx]

## Slide 1
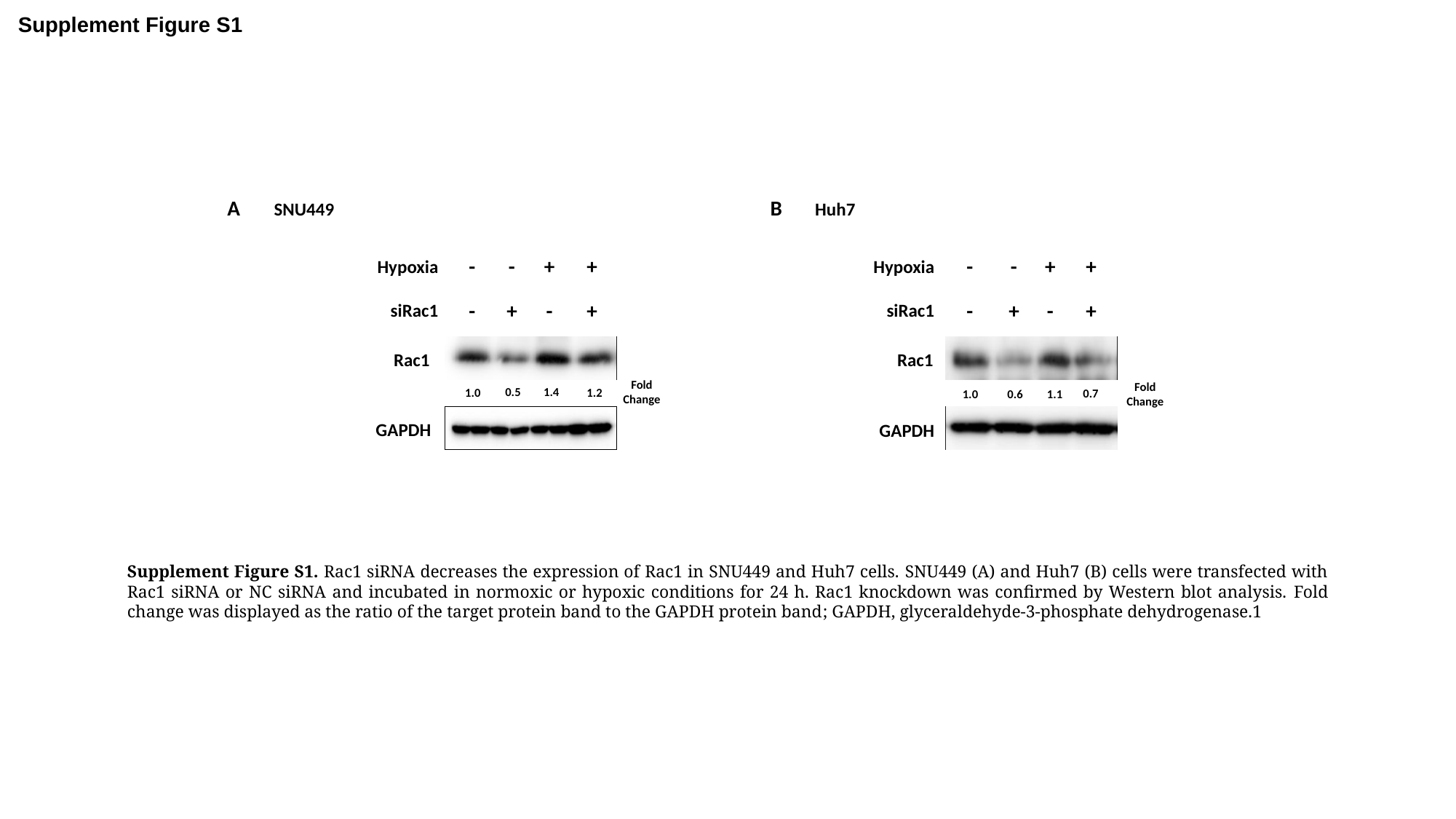

Supplement Figure S1
| | | | | | | | | | |
| --- | --- | --- | --- | --- | --- | --- | --- | --- | --- |
| Hypoxia | - | - | + | + | | | | | |
| siRac1 | - | + | - | + | | | | | |
| | | | | | | | | | |
| --- | --- | --- | --- | --- | --- | --- | --- | --- | --- |
| Hypoxia | - | - | + | + | | | | | |
| siRac1 | - | + | - | + | | | | | |
B
A
Huh7
SNU449
Rac1
Rac1
Fold
Change
Fold
Change
0.5
1.4
1.0
1.2
0.7
0.6
1.1
1.0
GAPDH
GAPDH
Supplement Figure S1. Rac1 siRNA decreases the expression of Rac1 in SNU449 and Huh7 cells. SNU449 (A) and Huh7 (B) cells were transfected with Rac1 siRNA or NC siRNA and incubated in normoxic or hypoxic conditions for 24 h. Rac1 knockdown was confirmed by Western blot analysis. Fold change was displayed as the ratio of the target protein band to the GAPDH protein band; GAPDH, glyceraldehyde-3-phosphate dehydrogenase.1
